# Periodic-net: an end-to-end data driven framework for diffuse optical imaging of breast cancer from noisy boundary data

**DOI:** 10.1117/1.JBO.28.2.026001

**Published:** 2023-02-06

**Authors:** Nazish Murad, Min-Chun Pan, Ya-Fen Hsu

**Affiliations:** a National Central University, Department of Mechanical Engineering, Taoyuan City, Taiwan; b Landseed Hospital International, Department of Surgery, Taoyuan City, Taiwan

**Keywords:** diffuse optical tomography, machine learning, optical imaging, oncology, inverse problem, frequency domain

## Abstract

**Significance:**

The machine learning (ML) approach plays a critical role in assessing biomedical imaging processes especially optical imaging (OI) including segmentation, classification, and reconstruction, intending to achieve higher accuracy efficiently.

**Aim:**

This research aims to develop an end-to-end deep learning framework for diffuse optical imaging (DOI) with multiple datasets to detect breast cancer and reconstruct its optical properties in the early stages.

**Approach:**

The proposed Periodic-net is a nondestructive deep learning (DL) algorithm for the reconstruction and evaluation of inhomogeneities in an inverse model with high accuracy, while boundary measurements are calculated by solving a forward problem with sources/detectors arranged uniformly around a circular domain in various combinations, including 16×15, 20×19, and 36×35 boundary measurement setups.

**Results:**

The results of image reconstruction on numerical and phantom datasets demonstrate that the proposed network provides higher-quality images with a greater amount of small details, superior immunity to noise, and sharper edges with a reduction in image artifacts than other state-of-the-art competitors.

**Conclusions:**

The network is highly effective at the simultaneous reconstruction of optical properties, i.e., absorption and reduced scattering coefficients, by optimizing the imaging time without degrading inclusions localization and image quality.

## 1 Introduction

Due to the excellent imaging capabilities, optical imaging (OI) techniques have been developed and employed to restore, segment, classify, and identify tissue properties.^1^^–^^4^ Diffuse optical imaging (DOI) is the one for being used in the reconstruction of optical properties of the brain and breast. Due to the growing power of artificial intelligence, many inverse problems in DOI are solved in deep learning-based algorithms to improve image reconstruction quality.^5^^–^^8^ Applications of ML in optical fields ranging from biotechnology^9^ to cancer diagnosis^10^ have shown great potential for localization, classifying, and segmentation of tumors for diverse samples in biomedical applications.^11^^–^^13^ Despite the excellent performance of convolution neural network (CNN)-based deep learning reconstruction methods, the locality of the convolution operator makes it difficult to learn global and long-range image information, i.e., reconstructing absorption and scattering coefficient in the same network well.^14^

Previously, numerous data-driven algorithms for sources and detectors have been used to reconstruct DOI optical-property images by generating simulation datasets for training.^13^^–^^17^ Some among those^16^ emphasize fast and accurate estimating of the bulk optical properties in the breast by exploiting a convolutional neural network. Various deep-learning approaches for numerous scientific domains and various forms of data analysis have been extensively reviewed for current work in optical properties retrieval.^18^^,^^19^ Well-controlled data sets for training and validation are among the most important topics in the neural network, but the lack of large, publicly available data sets leads to unique challenges. The development of a data generation pipeline^20^ based on Monte Carlo modeling has shown to be a useful method for rapid, robust, and user-friendly image formation in a wide variety of applications. Three-layer deep neural network is proposed in Ref. 21, and simulation, phantom, and clinical data with breast lesions are tested for the mismatch between the target and reference sides.

Some deep learning algorithms focused on restoring high-quality images by simultaneously considering both projection and image domains while the final images may suffer from secondary artifacts due to the introduced errors from projections interpolation.^22^ Zhang et al.^23^ present an encoder-decoder learning-based optimization network, which was highly effective in preserving inclusion edges and recovering details. Fortunately, domain transformation^24^ called AUTOMAP has the ability for reconstructing sensor information and shows good performance in optical image reconstruction tasks, while Dense-net,^25^ U-net,^26^ and its variants perform well in restoring directly from the image domain.

The primary contribution is that we first presented a network for DOI reconstruction called Periodic-net directly employed to boundary data, and then we modified state-of-the-art models, i.e., U-net and Dense-net for optical-property image reconstruction (these models are developed for the purpose of restoring biomedical images, not for the reconstruction of images). Additionally, our deep learning models include datasets that were previously unconsidered for state-of-the-art networks in DOI.

## 2 Materials and Methods

### 2.1 DOI Preprocessing and Postprocessing

**Fig. 1:**
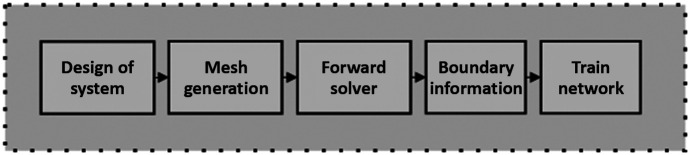
Flow chart of dataset(s) preparation for network training.

A unified formulation for the frequency domain (FD) system obtaining three combinations of boundary observations and conditions is adapted. Figure 1 illustrates the schematic of the preprocessing and postprocessing steps involved in the reconstruction of optical-property images. Light from laser modules is transmitted to the optical switch, which sequentially passes it to preselected points on the surface of the phantom to perform optical data acquisition. The boundary of phantom designated a source and detector positions. A simulation dataset based on FD measurements is generated using the finite-element forward solver, with Robin boundary conditions.^27^

#### 2.1.1 Signal generation in DOI

Signals are generated by photon radiance $L(r,\hat{s},t)$ in forward solver, where s^ denotes the direction of light r is position vector at time t. Photon radiance is calculated from radiation transport equation (RTE) in the presence of a nonlinear source term $Q(r,\hat{s},t)$ to characterize tissue by the scattering coefficient $\mu_{s}$ and the absorption coefficient $\mu_{a}$; formulation of optical-property is given as^28^

(1c∂∂t+s^.∇+μt)L(r,s^,t)=μs∫4πL(r,s^′,t)f(s^′,s^)ds^′+Q(r,s^,t), (1)

where c,f(s^′,s^) corresponds to the speed of light inside soft tissue and is a normalized phase function representing the probability of scattering, respectively. The transport coefficient is represented by $\mu_{t}$ to calculate the loss of radiance and is equal to the sum of reduced scattering ($\mu_{s}^{\prime }$) and absorption coefficients ($\mu_{a}$). Equation (1) can be further simplified and transformed into the frequency domain as

$$(-\nabla .(\frac{1}{3(\mu_{s}^{\prime }+\mu_{a})})\nabla +\mu_{a}-\frac{i\omega }{c})\Phi (r)=S_{0}(r),$$ (2)

where Φ(r), $S_{0}(r)$, and ω represent the photon density, source term, and frequency, respectively.

### 2.2 Dataset Preparation

#### 2.2.1 Generation of training samples

Training datasets are created by solving the forward problem and then optimization of the inverse problem is obtained through Tikhonov regularization (TR), applying Newton’s method to minimize the objective function,^29^ i.e.,

$$\underset{\Delta \chi }{\min}\{\|J\Delta \chi -\Delta \Phi {\|}_{2}^{2}+\lambda^{2}\|L\Delta \chi {\|}_{2}^{2}\}=\underset{\Delta \chi }{\min}\|\begin{array}{l} J\Delta \chi -\Delta \Phi \\ \lambda L\Delta \chi \end{array}{\|}_{2}^{2},$$ (3)

where L,λ are the dimensionless regularization matrix and the corresponding parameter of regularization of photon density. Δχdenotes fluence rate and J is Jacobian matrix.

To collect boundary data for the network, multiple excitations and measurement locations are employed; i.e., m source locations, equally spaced around the circular circumference, are assumed for each of the n excitation positions (n=m−1), thus yielding a total of m×n amplitude and m×n phase shift observations. This work employs three different simulation datasets that include boundary data from various measurements, i.e., system design for 16×15, 20×19, and 36×35 source–detector positions. A “noisy” boundary information obtained from the forward solver is delivered to the reconstruction network.

#### 2.2.2 Synthetic phantom data collection and calibration

The datasets with 16×15 measuring points (amplitude and phase shift) of each has been employed to perform DOI from laboratory experiments at our institute.^30^^–^^32^ By calibrating the data, it is possible to remove errors caused by model mismatches, such as detector responsivity, optical fiber differences errors, and numerical noise in the inverse computation of the heterogeneous medium. To calibrate the computed homogeneous data with the measured heterogeneous data, we performed calibrations using the computed homogeneous data. Optical-property coefficients assigned to a homogeneous phantom are used to compute homogeneous data.

#### 2.2.3 Simulation dataset for training

Three types of boundary data, i.e., 16×15, 20×19, and 36×35 were employed for training. Within this study, all 10,000 simulation samples for three boundary data types were prepared by our in-house computation code NIR•FD_PC.^27^^,^^33^^–^^35^ In addition, the code was also applied to reconstruct optical-property images ($\mu_{a}$ and ${\mu_{s}}^{\prime }$) using the TR method. A total of 4400 samples were formed with one inclusion and 5500 samples with two inclusions.^36^ The shapes of phantom background and its inclusion(s) for all samples were kept circular in the current study. The diameters of 80 phantoms range from 60 to 150 mm with their inclusions selected randomly. The driving frequencies of FD DOI for all samples were taken in the range 10 to 100 MHz. Background absorption and reduced scattering coefficients were chosen in the range 0.005 to $0.03\;{\mathrm{mm}}^{-1}$ and $0.05–3\;{\mathrm{mm}}^{-1}$, respectively, where ground truth images were formed directly from the parameters of inclusion in a rectangular grid. Datasets were then divided into three categories, i.e., training (85%), validation (10%), and testing (5%), respectively.

### 2.3 Deep Learning Framework

#### 2.3.1 Issues with traditional encoder–decoder network

**Fig. 2:**
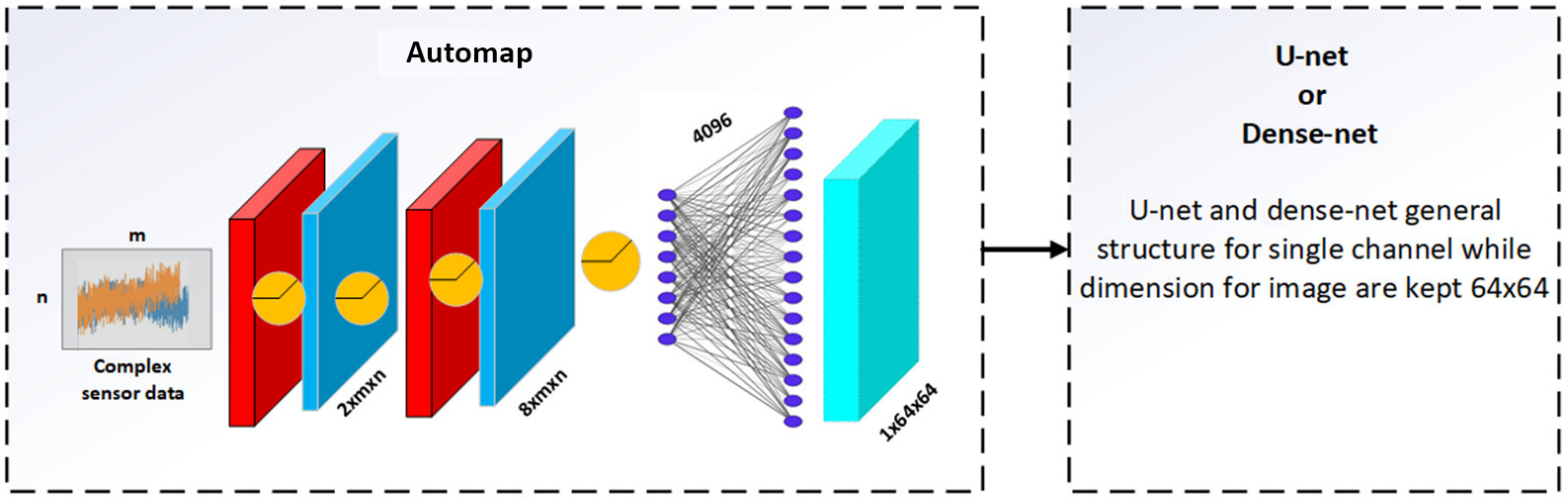
Modified U-net and Dense-net structures.

In our computation, we observed that traditional state-of-the-art machine learning encoder decoder methods failed to detect tiny structures in most cases (see later in the Results, Sec. 3). The reference networks are based on the encoder–decoder structure, i.e., U-net/Dense-net structure as the basic skeleton and AUTOMAP^24^ serves as the input layer (Fig. 2.).

### 2.4 Mechanisms of Periodic-net

**Fig. 3:**
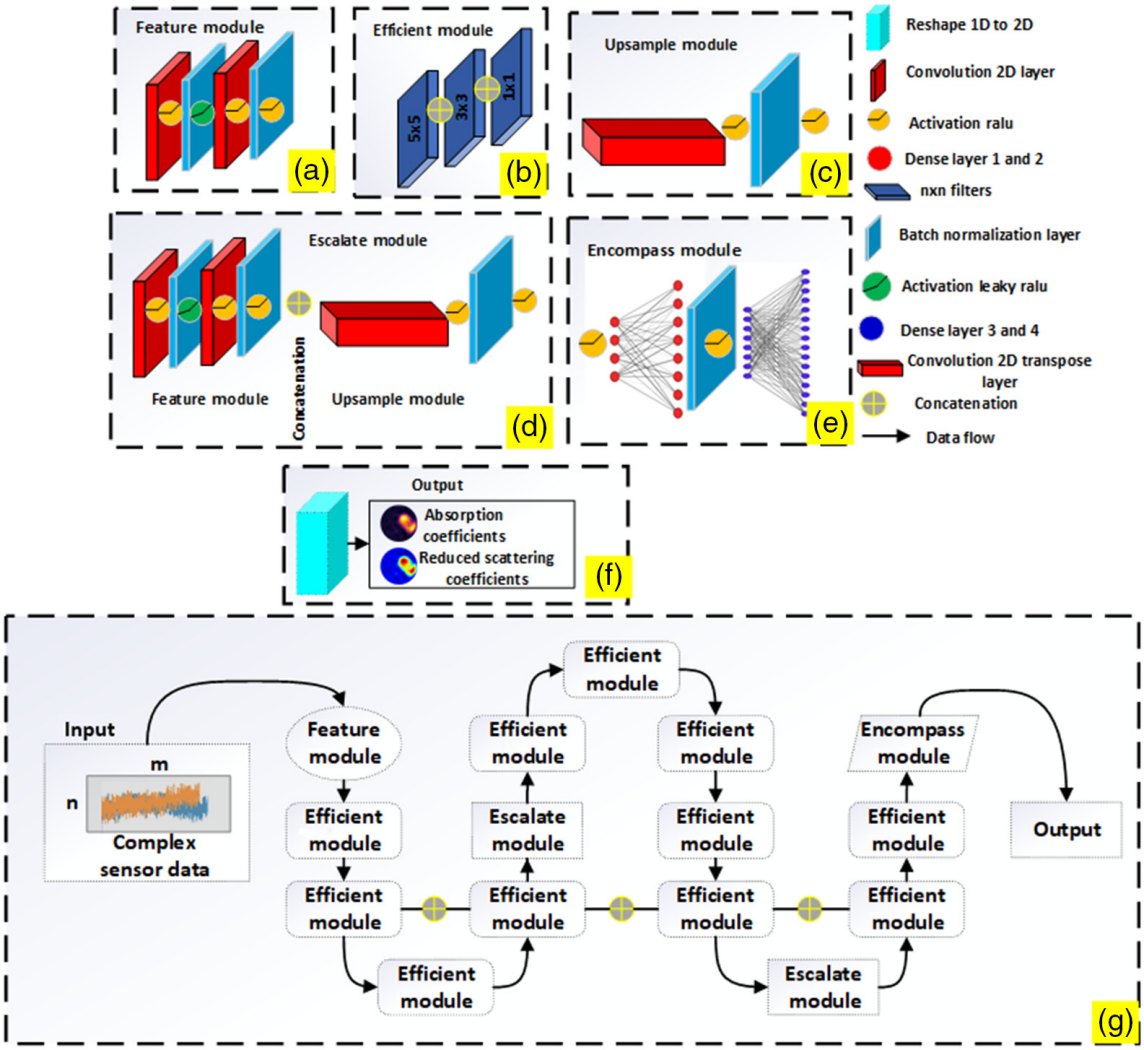
Flow of data in Periodic-net. (a) Feature module: consist of convolution layers. (b) Efficient module: extract tiny structure. (c) Upsample module: increasing dimension for (d). (d) Escalate module: concatenation of two modules. (e) Encompass module: reconstruction of optical images. (f) Output: reshaping to get absorption and reduced scattering coefficients. (g) Overall architecture of the proposed Periodic-net model. (a)–(d) Work as feature extraction; (e) working as reconstruction.

The overview of the proposed Periodic-net and its components are shown in Fig. 3. The overall architecture is based on four different modules performing individual tasks. The purpose is not only capturing small structures and reconstructing them but also to localize them in adequate resolution for detecting early-stage breast cancer. Feature module skeleton consists of two kinds of layers to extract features, i.e., convolution and batch normalization, each followed by activation function. The structure begins with two 3×3 eight-channel 2D convolutional layers, followed by a batch normalization and ReLU activation layer. Convolution is performed on an input volume, batch normalization is utilized, and then ReLU activation is applied in each feature block. The inside structure of feature module is shown in Fig. 3(a). Four feature blocks are instantiated in an efficient module. In the first feature block, 1×1 convolution was applied, followed by 3×3, and 5×5 filters with the same padding, which ensures that the output volume sizes are identical to all blocks and the maximum information is extracted from limited neurons since the input has a smaller number of neurons as compared with the output. A channel dimension is used to concatenate the output volumes. A major objective of the efficient module is to assess how locally sparse structures can be approximated [Fig. 3(b)]. Input volumes are reduced by the escalate module. This module utilizes two branches in a similar manner to the efficient module. First, a 3×3 convolution is performed, but with a stride of 2×2 and valid padding, resulting in a reduction in volume size. In the second branch, 3×3 maximum pooling is used with a 2×2 stride. Then it is possible to concatenate the output volumes for both branches along the channel axis [Fig. 3(d)]. Image reconstruction is performed in encompass module. Due to the low contrast between background and inclusions, we employ dense layers to extract more discriminative features. Four dense layers are in the encompass module, each followed by a batch normalization layer and an activation of the ReLU layer. Dense layers are composed of 64, 128, 256, and 4096 neurons at the bottom. The final output size is 1×64×64 for the absorption coefficient and 1×64×64 for the reduced scattering coefficients [Fig. 3(e)]. While Fig. 3(f) shows expected output for circular phantom samples. During training, the periodic nature of proposed network is shown in Fig. 3(f); apart from that, concatenation between two efficient modules are performed to avoid degradation problems.

### 2.5 Network Configuration and Training

The simulation dataset consists of a total of 10,000 samples, which are divided into training, validation, and test sets according to a distribution of 8500, 1000, and 500, respectively.^6^ Adam optimizer is set to 0.001 and is used as gradient updating; other parameters include momentum of 0.5, batch size of 64, and weight decay of $10^{-4}$. A quantitative evaluation of reconstruction results is conducted using the mean square error (MSE),^37^ peak signal-to-noise ratio (PSNR),^38^ and structural similarity index (SSIM).^39^ We trained all models for a total of 20 epochs, in which

$$\mathrm{PSNR}=10\;\log_{10}\frac{\max^{2}(x)}{\mathrm{MSE}},$$ (4)

is the PSNR that calculates the difference between corresponding pixels and the maximal intensity value; the MSE is for the mean square error during training

$$\mathrm{SSIM}=\frac{\left(2x_{\mathrm{true}}x_{\mathrm{DOT}}+\mathrm{const}\_1)(2\sigma_{\mathrm{true},\mathrm{DOT}}+\mathrm{const}\_2\right)}{\left(x_{\mathrm{true}}^{2}+x_{\mathrm{DOT}}^{2}+\mathrm{const}\_1)(\sigma_{\mathrm{True}}^{2}+\sigma_{\mathrm{DOT}}^{2}+\mathrm{const}\_2\right)},$$ (5)

where SSIM evaluates the structural similarities between reconstructed diffuse optical tomography (DOT) and ground truth, $x_{\mathrm{true}}$, $x_{\mathrm{DOT}}$ are true and reconstructed optical properties, i.e., absorption and reduced scattering coefficients, and $\sigma_{\mathrm{true}},\sigma_{\mathrm{DOT}}$ are the means and variances of reconstructed images of absorption and reduced scattering coefficients. The positive constants $\mathrm{const}\_1=0.01^{2}$, $\mathrm{const}\_2=0.02^{2}$ avoid a null denominator.

## 3 Results

In this section, three independent versions of the datasets were trained for the same network, responsible for DOI images of breast cancer. We conduct a series of simulations and experiments confirming and evaluating the performance of the proposed network described in detail in Sec. 2.4. Initially, we present simulated and phantom experiment results that demonstrate the image formation process works both with 16 source positions and 15 detector positions. The second step involves evaluating the reconstructed images from simulated data by varying the number of sources and detectors, i.e., 20×19 and 36×35. Selective test sample parameters and other specifications are given in section Table 1. A side-by-side comparison between different algorithms is shown. Randomly generated Gaussian noise of 15% is added to the forward problem to mimic the noise and aberrations that might arise in phantom test set due to experimental hardware. The fluence rate values were normalized using the min-max method to fix all the values between 0 and 1.

**Table 1:** Random cases from test datasets for verification.

Dataset	Frequency (MHz)	Phantom diameter (mm)	Inclusion(s) radius (mm)
16×15	20	50	5
	10	70	11.25, 6.77
20×19	50	85	10.62, 5.27
36×35	80	80	10.39, 5.93

**Fig. 4:**
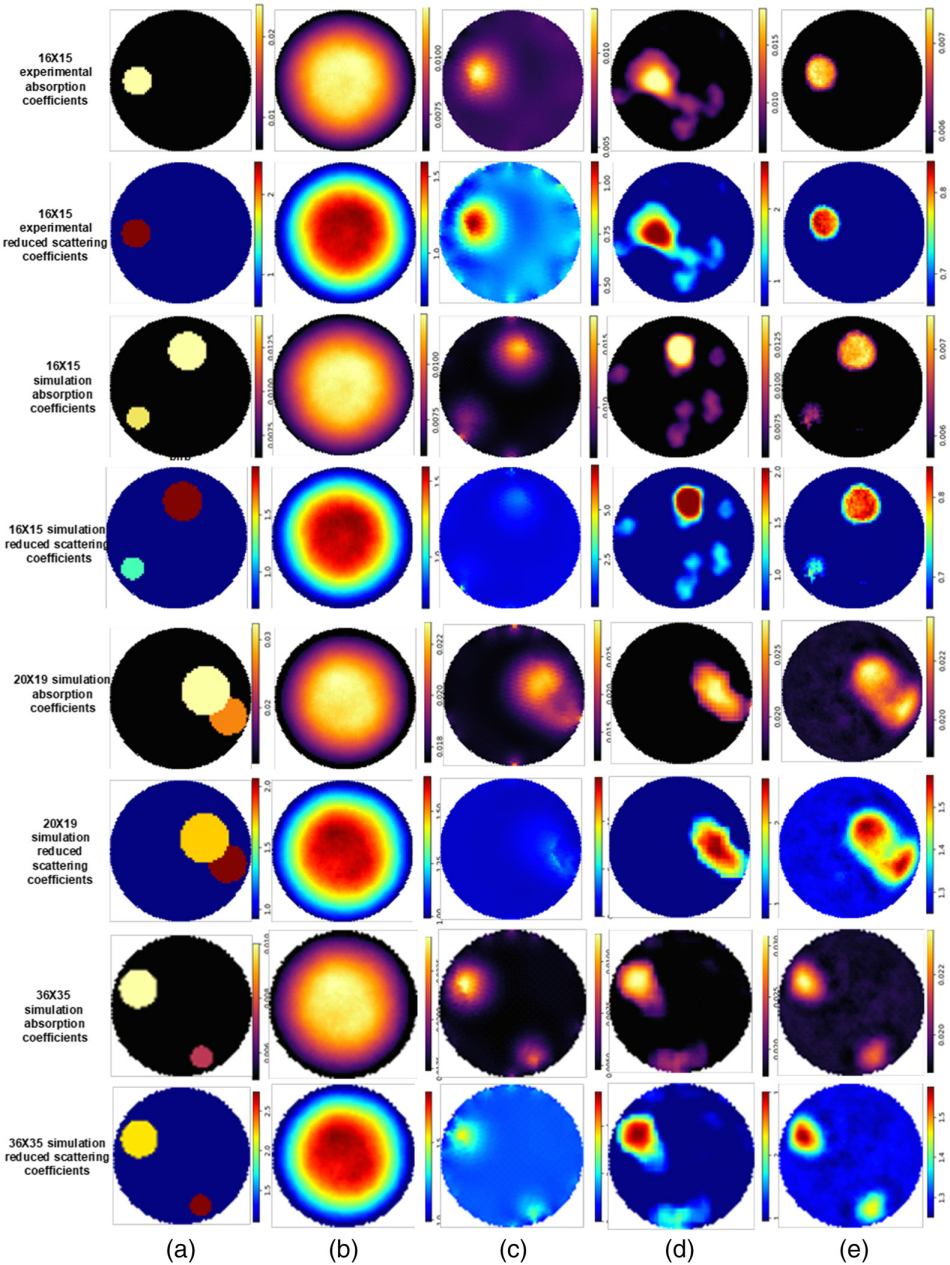
Phantoms with different sizes, using one and two inclusions. (a) Ground truth. (b) Dense-net. (c) TR. (d) U-net. (e) Periodic-net. Overall, Periodic-net and U-net perform well to reconstruct a better shape and size of inclusion(s) with less noise.

The reconstructed images in the two-dimensional plane from all methods are shown in Fig. 4. Absorption and reduced scattering coefficients are plotted from upper row to lower row according to the dataset while each column represents reference and proposed methods: the first column is for ground truth, the second column for Dense-net, the third column represents traditional iterative Tikhonov regularization, the fourth column specify U-net, and the last column reconstruct optical properties via Periodic-net.

### 3.1 Dataset 1: Phantom Case with 16 × 15 Boundary Data

Two different experimental samples were carried out from the test dataset with one and two inclusions, respectively. We have performed laboratory phantom studies to confirm what we found. However, a finer reconstruction can be seen in the first experiment, in which a single inclusion is embedded into a medium (phantom) with an absorption coefficient $\mu_{a}=0.0059\;{\mathrm{mm}}^{-1}$ at a frequency 20 MHz, and to match the background scattering coefficient of $0.69\;{\mathrm{mm}}^{-1}$. The target is placed near the boundary [Fig. 4 (first – fourth rows)]. In the second experiment, two targets were embedded, which were 11.25 and 6.77 mm diameter spheres. The diffusion is measured for both samples in a circular domain using a DOT photon array system. We demonstrate that the U-net model trained with 16×15 simulation measurements significantly outperforms the Dense-net model trained with either the simulation absorption coefficients or reduced scattering coefficients images. It should be noted that U-net-based methods produce erroneous boundaries for large structures, especially when the boundaries are blurry, such as those depicted in Fig. 4(d). U-net can localize structure representations in larger areas more effectively than in smaller structures. A blurred reconstructed image can be observed for TR, whereas a clear reconstructed image of one inclusion and two inclusions with an accurate shape is found using U-net. In contrast, Periodic-net has a high degree of precision in detecting tiny structures.

### 3.2 Dataset 2: Phantom Case with 20 × 19 Boundary Data

Figure 4 (rows 5 and 6) shows a reconstruction of optical properties from 20×19 measurements, i.e., 19 detector positions around phantom at wavelengths λ=5.99  m during absorption coefficients and similar for reduced scattering coefficients. The data from all source-detector position pairs for the diameter range from 60 to 150 mm as input to our reconstruction network. The FEM model consisted of a circular mesh with a radius of 10.62 and 5.27 mm. However, it is evident that Dense-net [Fig. 4(b)] could not produce scattering and absorption coefficient images with fine structure, and the respective values of different locations of imaging target(s) were underestimated. Periodic-net improves resolution than U-net and TR. We observe that the Dense-net gives the worst results as they do not reconstruct any inclusion. U-net gives slightly better results than the TR but is still unsatisfactory.

### 3.3 Dataset 3: Phantom Case with 36 × 35 Boundary Data

In this section, the sample under consideration belongs to 36 source positions and 35 detector positions that are arranged on the surface of the phantom uniformly. Therefore, there are a total of 36×35=1260 observations. In this sample, we considered an inhomogeneous medium with two inclusions (Fig. 4, rows 7 and 8). The origin of the coordinates for the first inclusion is set at the left bottom near the center and the second inclusion is set at the right upper half near the center of the surface. A set of absorption and reduced scattering coefficients of $\mu_{a}=0.0080\;{\mathrm{mm}}^{-1}$ and $\mu_{s}=0.5634\;{\mathrm{mm}}^{-1}$ are selected to simulate the background tissue. To quantify the comparison among the four methods, we have reconstructed the absorption and scattering coefficient separately from the regularization method [Fig. 4(c)] and for two state-of-the-art networks [Figs. 4(b) and 4(d)]. It is seen that there are significant improvements in reconstructed image values with Periodic-net, as we expected, while those from U-net are better than those from Dense-net. It is clear from Fig. 4(e) that in the proposed method leads to an enhanced reconstructed contrast, thus resulting in clearer DOT images. The reconstruction results of the proposed Periodic-net are the closest to the ground truth compared with those of the TR, modified U-net, and modified Dense-net models. The proposed model is helpful to recover the spatial information that is lost during the iterative method [Fig. 4(c)].

## 4 Discussion

This study proposed a DL model, called Periodic-net, aiming for better imaging of breast tumors and improving the overall performance in terms of spatial resolution, reconstruction time, and memory storage. A greater number of source positions around the phantom has the advantage of improving the reconstruction of absorption and scattering coefficients. However, it costs more during the reconstruction time. Based on our results, the Periodic-net is capable of learning features efficiently even for a small number of measurements. In order to make sure that the trained network enables us to measure the correct contrast for various measurements, we used a variety of contrasts in the training process.

Four key modules make up the overall structure of a Periodic-net: feature module, efficient module, escalate module, and encompass module. In the feature module, raw data (fluence rate) are transformed into numeric features that can be processed while preserving the information in the original data set. Then, an end-to-end edge enhancement reconstruction subnetwork reconstructs the initial image with sparse artifacts removal and image edges preservation in the efficient module. However, the transforming domain may introduce unexpected artifacts. Therefore, the data escalate module, which consists of two submodules (one for feature extraction, and the other for domain correction), was introduced to reduce errors, ensure consistency, and improve structural details. A submodule (upsample module) for edge enhancement is introduced to preserve features and reduce blurring along the edge of the tomographic image. Finally, encompass module is introduced to capture global features from the efficient module and reconstruct the structure of optical properties, i.e., absorption and scattering coefficients.

U-net and Dense-net cannot be applied to DOI directly, so modification was made to these two nets to reconstruct optical properties in different domains. Both these state-of-the-art networks have shown good performance on semantic segmentation, object detection, and classification with localization. There is a slight improvement in U-net performance over Dense-net in terms of DOI reconstruction. It is worth noting that both networks are designed to take high-resolution images as inputs. The U-net architecture showed good performance on the one inclusion data while failing to detect and construct small lesions. Since the U-net utilizes a completely convolutional architecture, only high-level features are considered as a result. While Dense-net has more layers and hyperparameters than U-net, it still fails to extract any useful information such as edges or localizations. Careful arrangement of layers and cautious selection of hyperparameters are crucial to the reconstruction of optical-property images.

Our architecture retrieves optical properties images of breast lesions from low-contrast DOI. Furthermore, it does not rely on the number of radiances nor on the domain, i.e., multiple excitations and measurement positions are used to produce the boundary data. Figure 4 illustrates the reconstruction results of single and two inclusions cases for different boundary measurements. For all simulations 16, 20, and 36 excitation positions and 15, 19, and 35 measurement locations (both are equally spaced around the circular circumference in angular increments beginning near 0 deg, depending on local meshing details). It is also important to note that different modules of network structures and loss functions are designed and implemented at different stages during the training process. In addition to achieving good performance, we also achieve the benefits of fewer parameters and faster convergence (∼3  s) (Table 2). We demonstrate an approach that enables noninvasive OI behind scattering photons in breast cancer tissue. Experimental validation shows the efﬁciency and robustness of the method with various DOT samples, covering a reconstruction of up to three different datasets w.r.t boundary data.

**Table 2:** Efficiency comparison between trained Periodic-net with different networks using three datasets.

	Dense-net	U-net	Periodic-net
Boundary data 16×15			
Training time(s) per epoch (s)	31	20	3
Trainable parameters	18,292,788	17,681,797	1,258,308
Nontrainable parameters	3140	8212	9892
Memory (kb)	215,380	207,542	15,766
Boundary data 20×19			
Training time(s) per epoch (s)	33	28	3
Trainable parameters	22,880,308	26,856,837	1,310,532
Nontrainable parameters	3140	8212	9892
Memory (kb)	269,140	315,062	16,378
Boundary data 36×35			
Training time(s) per epoch (s)	45	32	∼3
Trainable parameters	51,716,148	43,240,957	1,642,308
Nontrainable parameters	3140	8212	9892
Memory (kb)	607,060	507,059	20,266

The superiority of the method compared with conventional techniques is shown by applying it to DOT problems of different breast cancer data of a various number of measurement data. In contrast to traditional segmentation, classification, and super-resolution imaging approaches, our method does not utilize medical images directly for training to attain optical properties, but rather focuses on reconstruction and improving image quality after changing sensor data into images in one step, which is an end-to-end model. Using inhomogeneous cases, we demonstrate that our method can also be applied to nonsparse and continuous objects. We note that a few iterations are sufﬁcient to recover the object reasonably well in our case. In addition, our technique is not limited by the number of independent illuminations/fluence rates that can be generated with one source/detector, since the scattering angle of the illumination can be tuned to produce an extremely large number of independent illuminations with different positions. Additionally, calibrated measure data have been used to increase the number of test samples and check the impact of overfitting.

According to test results, U-net and TR are still unable to reconstruct small anatomical landmarks with blurred borders. Although U-net is good at reconstructing large structures, it fails when the inclusions are small or have noisy boundaries which can be seen in Fig. 4. Results using simulated data suggest that qualitative images can be produced that readily highlight the location of absorption and scattering heterogeneities. Our proposed (Periodic-net) modeling approach was found to yield the best results after comparing various convolutional neural network architectures, i.e., U-net, and Dense-net, as well as the conventional regularization approach, i.e., TR.

**Fig. 5:**
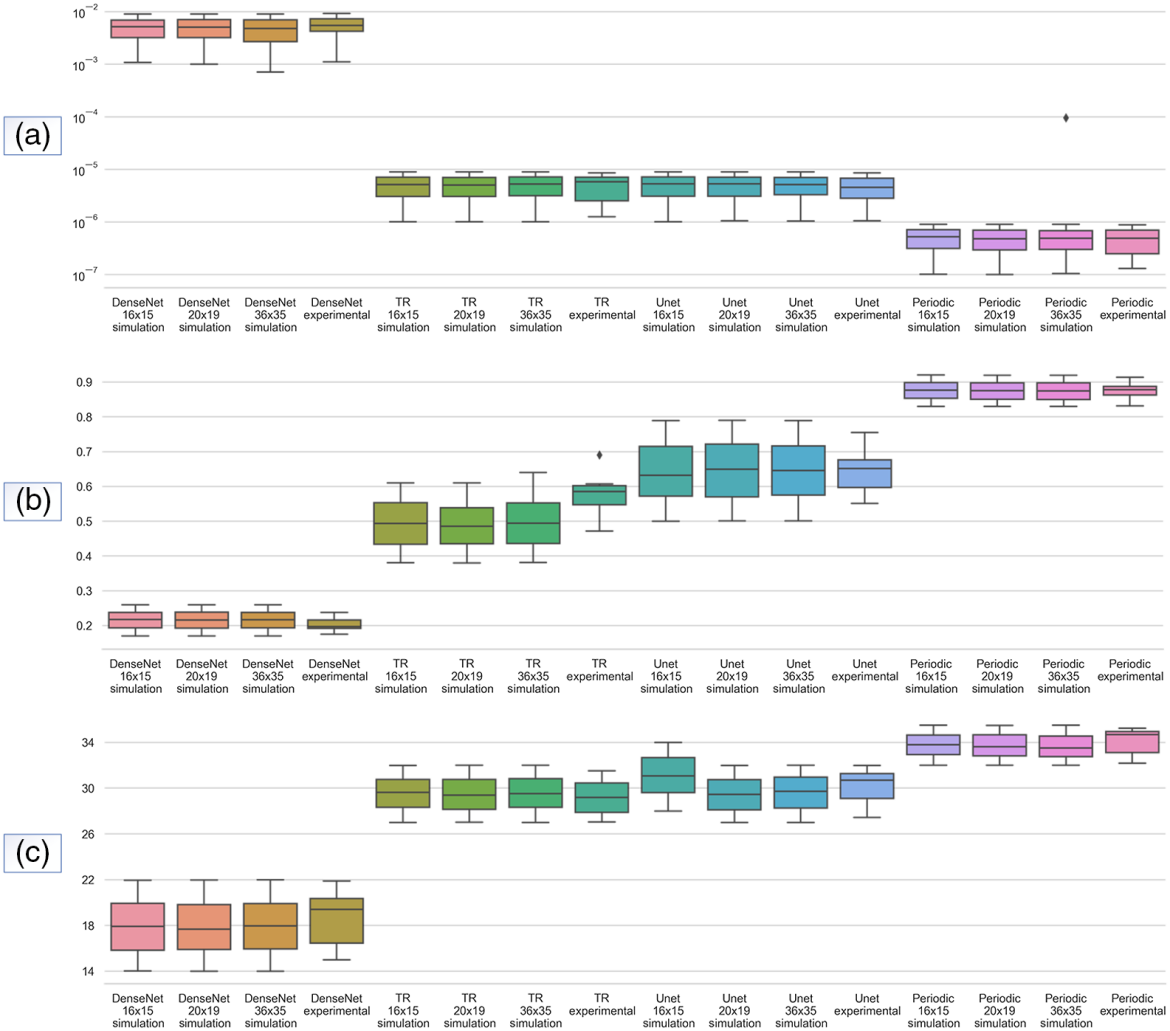
Overview of statistical results for reduced scattering coefficients from (a) MSE, (b) SSIM, and (c) PSNR. 500 samples from simulation datasets of 16×15, 20×19, and 36×35 boundary data, and 12 samples from 16×15 experimental dataset are presented.

**Fig. 6:**
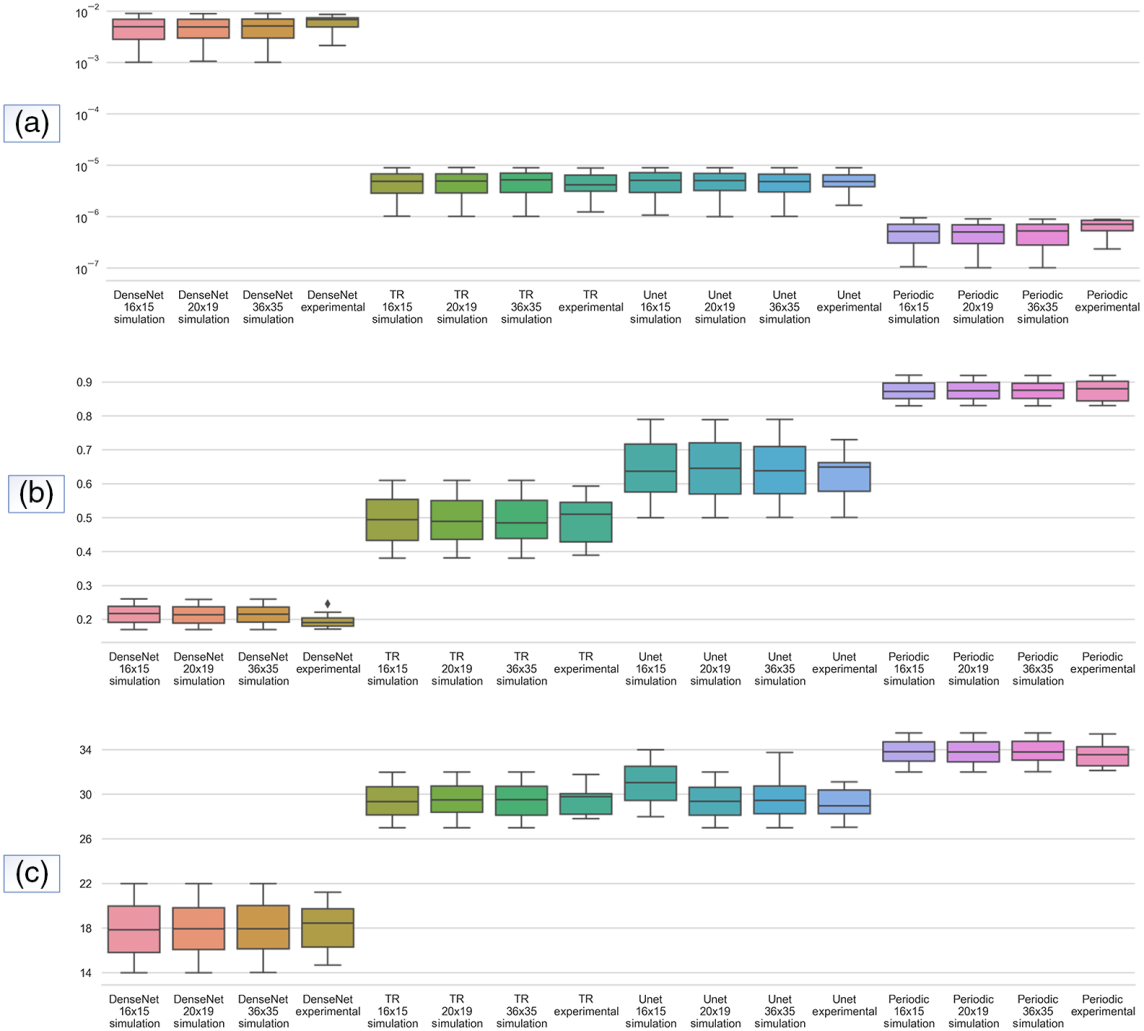
Overview of statistical results for absorption coefficients from (a) MSE, (b) SSIM, and (c) PSNR. 500 samples from simulation datasets of 16×15, 20×19, and 36×35 boundary data, and 12 samples from 16×15 experimental dataset are presented.

A quantitative evaluation of all methods was also conducted. Figures 5 and 6 represent the results for the reduced scattering coefficients and absorption coefficients, respectively. The MSE, PSNR, and SSIM metrics were calculated to verify the effectiveness of the edge enhancement reconstruction. A significant improvement in the absorption coefficients can be observed, where the Periodic-net model has the lowest MSE value. The results of the Periodic-net showed a significant reconstruction improvement compared with the other models, with a maximum SSIM score of 0.89 for absorption coefficients. Likewise, for the reduced scattering coefficients the Periodic-net model achieved the highest PSNR. Overall, the proposed Periodic-net model outperformed the TR, modified U-net, and modified Dense-net in terms of the evaluation of SSIM and PSNR scores. This shows the power of the proposed model to learn complicated features through various module connections in the proposed Periodic-net, which take advantage of the learning features directly from boundary data as input. The SSIM and PSNR scores show that the Dense-net performed worst, and U-net achieved better results than Dense-net. However, because of its periodic nature (cycle repeats in equal intervals of times for each epoch), Periodic-net outperformed TR and learning approaches in all evaluations. The Periodic-net has the smallest MSEs and the largest PSNRs and SSIMs as compared with other competitors.

## 5 Concluding Remarks

Periodic-net offers a potential reconstruction of soft tissue optical coefficients that are not only cost-effective, sensitive, and noninvasive but also provide better localization and suppresses noise when compared with existing state-of-the-art networks. From the simulation and experimental samples, it has been demonstrated that the proposed algorithm has not only remarkably improved the predicted accuracy and resolutions well as significant improvement in performance and reduction in testing and reconstruction time. The future directions of our work include extending our method to explore the feasibility of our Periodic-net with ring system-type datasets for more than one source and detector in different organizations.

## Code, Data, and Material Availability

Code and data will be made available on request.
